# Parametric Mapping of Brain Tissues from Diffusion Kurtosis Tensor

**DOI:** 10.1155/2012/820847

**Published:** 2012-08-29

**Authors:** Yuanyuan Chen, Xin Zhao, Hongyan Ni, Jie Feng, Hao Ding, Hongzhi Qi, Baikun Wan, Dong Ming

**Affiliations:** ^1^Department of Biomedical Engineering, Tianjin University, Tianjin 300072, China; ^2^Department of Radiology, Tianjin First Center Hospital, Tianjin 300192, China

## Abstract

Diffusion kurtosis imaging (DKI) is a new diffusion magnetic resonance imaging (MRI) technique to go beyond the shortages of conventional diffusion tensor imaging (DTI) from the assumption that water diffuse in biological tissue is Gaussian. Kurtosis is used to measure the deviation of water diffusion from Gaussian model, which is called non-Gaussian, in DKI. However, the high-order kurtosis tensor in the model brings great difficulties in feature extraction. In this study, parameters like fractional anisotropy of kurtosis eigenvalues (FAek) and mean values of kurtosis eigenvalues (Mek) were proposed, and regional analysis was performed for 4 different tissues: corpus callosum, crossing fibers, thalamus, and cerebral cortex, compared with other parameters. Scatterplot analysis and Gaussian mixture decomposition of different parametric maps are used for tissues identification. Diffusion kurtosis information extracted from kurtosis tensor presented a more detailed classification of tissues actually as well as clinical significance, and the FAek of *D*-eigenvalues showed good sensitivity of tissues complexity which is important for further study of DKI.

## 1. Introduction

Diffusion magnetic resonance imaging can detect the water molecule diffusion in human tissues noninvasively, which indicates the microstructure of biotissue such as one of the most popular methods: diffusion tensor imaging (DTI), in which the three-dimensional water diffusion probability distribution in an anisotropic medium has been quantified by a 2-ranked tensor. The three eigenvectors of it are corresponded to the axes of a triaxial diffusivity ellipsoid [1]. The commonly employed rotationally invariant parameters are derived from the diffusion tensor (DT) including the mean diffusivity (MD) and fractional anisotropy (FA).

However, in the conventional diffusion tensor imaging (DTI), water diffusion is assumed that diffusion appears to be a free and nonrestricted environment within a Gaussian distribution of diffusion displacement. Actually in biological tissue, complex cellular microstructure makes water diffusion a highly hindered or restricted and also non-Gaussian process [2]. The diffusion tensor loses many details of the tissues microstructure. There are many studies about the non-Gaussian of diffusion in real tissues. DKI uses the kurtosis to estimate this non-Gaussian distribution providing insights into the microstructure of biological tissues. Recent studies have demonstrated that DK (diffusion kurtosis) measures offer an improved sensitivity in detecting developmental and pathological changes in neuronal tissues, compared to conventional DTI [3, 4]. In addition, directional kurtosis analyses have been formulated to reveal directionally specific information, such as the water diffusional kurtosis along the direction parallel or perpendicular to the principle water diffusion direction [5–8]. Because kurtosis is a measure of the deviation of the diffusion displacement profile from a Gaussian distribution, DKI analyses quantify the degree of diffusion restriction or tissue complexity.

However, what is difficult is that the high-order (3-dimensional 4-order) kurtosis tensor in DKI is complex to analyses and it is an important feature like the diffusion tensor in DTI (diffusion tensor imaging). This paper here is to promote some parameters mapping of the kurtosis tensor.

## 2. Materials and Method

### 2.1. Theory

#### 2.1.1. Diffusion Kurtosis Imaging

As DTI assumes Gaussian diffusion, the apparent diffusivity (*D*_app_) is derived by linearly fitting the DW signals acquired with one or more nonzero *b* values to the following linear equation:

(1) $$\begin{array}{c} \ln\;\left[\frac{S\left(b\right)}{S\left(0\right)}\right]=-bD_{\text{app}}. \end{array}$$

In DKI, logarithmic expansion of DW signal can estimate both apparent diffusivity (*D*_app_) and diffusion kurtosis (*K*_app_), which keep an extra *b*-square term compared with DTI. Thus, it forms a nonlinear equation [8]:

(2) $$\begin{array}{c} \ln\;\left[\frac{S\left(b\right)}{S\left(0\right)}\right]=-bD_{\text{app}}+\left(\frac{1}{6}\right)b^{2}{D_{\text{app}}}^{2}K_{\text{app}},\\ D_{\text{app}}=Dx^{2},\\ K_{\text{app}}={\left(\frac{\text{MD}}{D_{\text{app}}}\right)}^{2}Wx^{4}, \end{array}$$

where *S*(*b*) is the DW signal intensity at a *b* value and *S*(0) is the signal without diffusion gradient; *x* is the gradient magnetic encoding directions; MD is the mean diffusivity; here appear the diffusion tensor (*D*) and kurtosis tensor (*W*), which characterize the different diffusion motion. The theory also indicates that the apparent diffusional kurtosis approaches the true diffusional kurtosis in the limit of short gradient pulse durations, which is analogous to the relationship between *D*_app_ and the true water diffusion coefficient *D*. 

#### 2.1.2. *D*-Eigenvalues of Kurtosis Tensor

DKI model provides a high-order tensor except a two-order diffusion tensor, so that the problem becomes more knotty. Just like the eigenvalues and eigenvectors of diffusion tensor from which we can easily get a visualized structure model, and also more insights into the tissues microstructure, which is viable and potential via this 4-order 3-dimensional fully symmetric tensor, diffusion kurtosis tensor (DK).

*D*-eigenvalues of the DK were proposed mathematically with an assumption that  *D*  tensor is always positive definite [9]. Then, a conversion was used:

(3) $$\begin{array}{c} D^{-1}\left(Wx^{3}\right)=\lambda x,\\ Dx^{2}=1. \end{array}$$

A number of  *D*-eigenvectors are obtained using the Sylvester formula of the resultant of a two variable system. The  *D*-eigenvalues values are

(4) $$\begin{array}{c} \lambda_{i}=Wx_{i}^{4}. \end{array}$$

### 2.2. Data Processing

#### 2.2.1. Data Acquisition

The whole study and all the human experiments have got the medical ethics authentication and each subject or volunteer has knew it clearly. All human experiments were conducted on a Siemens 3. 0 T Scanner System with the physicist and 20 volunteers were normal adults in the age between 20 and 30. The DW data were acquired with SE-EPI (single shot echo-echo planar imaging) sequence, following 30 gradient magnetic encoding directions and three *b* values (0, 1000, 2000 ms/*μ*m^2^). Additional image parameters were that image orientation is transverse, TR = 10500 ms, TE = 103 ms, average = 1, TA = 11′14′′, noise level = 30, acquisition matrix = 128 × 128, FOV = 230 × 230 mm^2^, slice thickness = 1.8 mm, number of slices = 73, no gap. 

For each subject a 128 × 128 × 73 × 61 metric data was acquired and prepared to fitting the tensors. Imaging processing including eddy current correction and 3D motion correction was conducted with FSL software. And for accessibility the normalization to Standard brain was used under SPM. Then the *D*_app_ and *K*_app_ were fitted using LMS method and LS method to estimate the optimal components of tensors.

#### 2.2.2. Parameters Mapping

Although there is a way to analyse the high kurtosis tensor, the number of  *D*-eigenvalues is random within the range between 3 and 13, even the practical meaning of these vectors and values is not clear. Anyhow, some parameters can be acquired for a further research.

In DKI, both DT and KT are obtained, and some kurtosis parameters can be formed following DT's method. Then the DTI- & DKI-derived parametric maps were analysed on the contrast between different tissues. Here are  *vD*-eigenvalues (*λ*_*i*_) or  *D*-eigenvectors (*x*_*i*_) from kurtosis tensor, FAek (FA about eigenvalues of kurtosis tensor), Mek (mean eigenvalues of kurtosis tensor), AKC (apparent kurtosis coefficient), AKCd (apparent diffusion kurtosis coefficient) are defined as following:

(5) $$\begin{array}{c} \text{FAek}=\surd \left(\frac{v}{\left(v-1\right)}\right)\cdot \surd \left(\frac{\left({\sum \left(\lambda_{i}-\text{Mek}\right)}^{2}\right)}{\left(\sum {\lambda_{i}}^{2}\right)}\right)\\ \text{Mek}=\text{mean}\left(\lambda \right)\\ \text{MK}=\text{mean}\left(K_{\text{app}}\right)\\ \text{AKCd}=\text{AKC}\cdot \text{M}{\text{D}}^{2}. \end{array}$$

**Figure 1:**
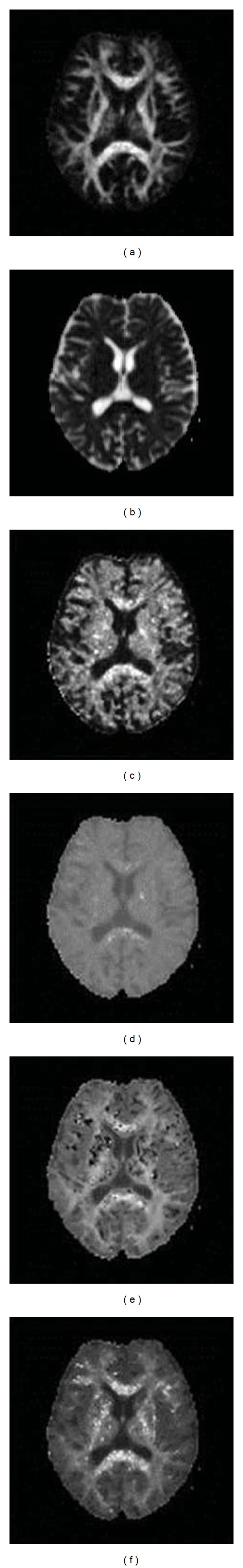
Parametric mapping from a same anatomical slice, ((a)–(f)) FA, MD, FAek, Mek, MK, and AKCd.

In the mapping of MK, these negative *K*_app_ values are revised as zeros as no medical significance. And for comparison, we also get AKC (mean of *K*_app_) without revising. Except these parameters above, this paper also considers FA (fractional anisotropy) and MD (mean diffusivity) from DTI. And that the MK (mean kurtosis) [6, 7] is considered as its popular use.  Figure 1 shows some parametric maps.

#### 2.2.3. Data Analysis

**Figure 2:**
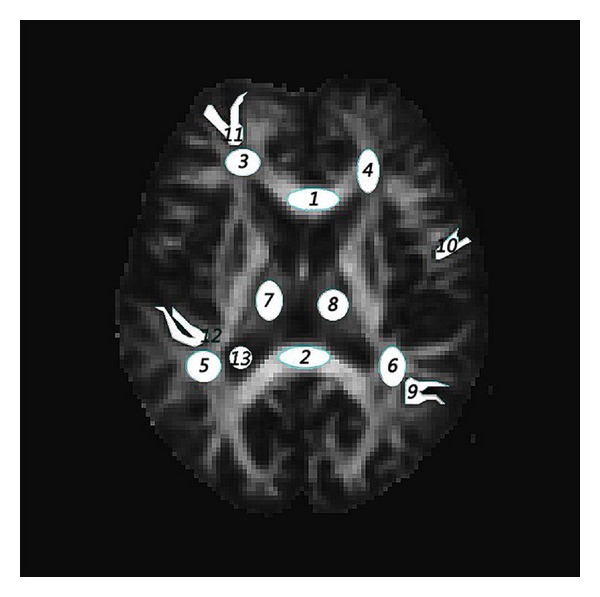
The ROIs selection according FA map. (1-2) the knee and splenial of callosum; (3–6) the crossing fibers; (7-8) the thalamus; (9–12) the cerebral cortexes; 13 is CSF.

For each subject, regions of interest (ROIs) were manually defined in several transverse slices by referencing to the anatomical structure. Anatomical landmarks were identified from both FA and MD. 6 WM structures were chosen, including the knee and the splenium of corpus callosum (CC); 4 crossing fiber areas, 4 of which are the extend areas along the knee and the splenium of CC and where are full of crossing fibers; 2 areas of thalamus; 6 GM structures, namely, 4 cerebral cortex CSF, more details from Figure 2. Various properties such as diffusivity or kurtosis can help to recognize the tissues [10, 11].

The mean and standard-variance were computed by volume averaging within the multislice ROIs for each structure. For each parameter, analysis of variance (ANOVA) was performed to compare the measurements among different tissues, followed by independent-samples  *t*-test to detect intergroup differences. 

Also in order to combining the whole image and the relativity between each other, we scattered two of the parameters with their gray histogram curves. As we already know that same tissues' voxel gray values' distribution is similarly Gaussian, and that we can assume that the histogram curves a first-order Gaussian mixed signal. The decomposition may be some single independent parameter histogram curve and also a scattered figure.

## 3. Results

### 3.1. Results of ROIs Analysis

**Figure 3:**
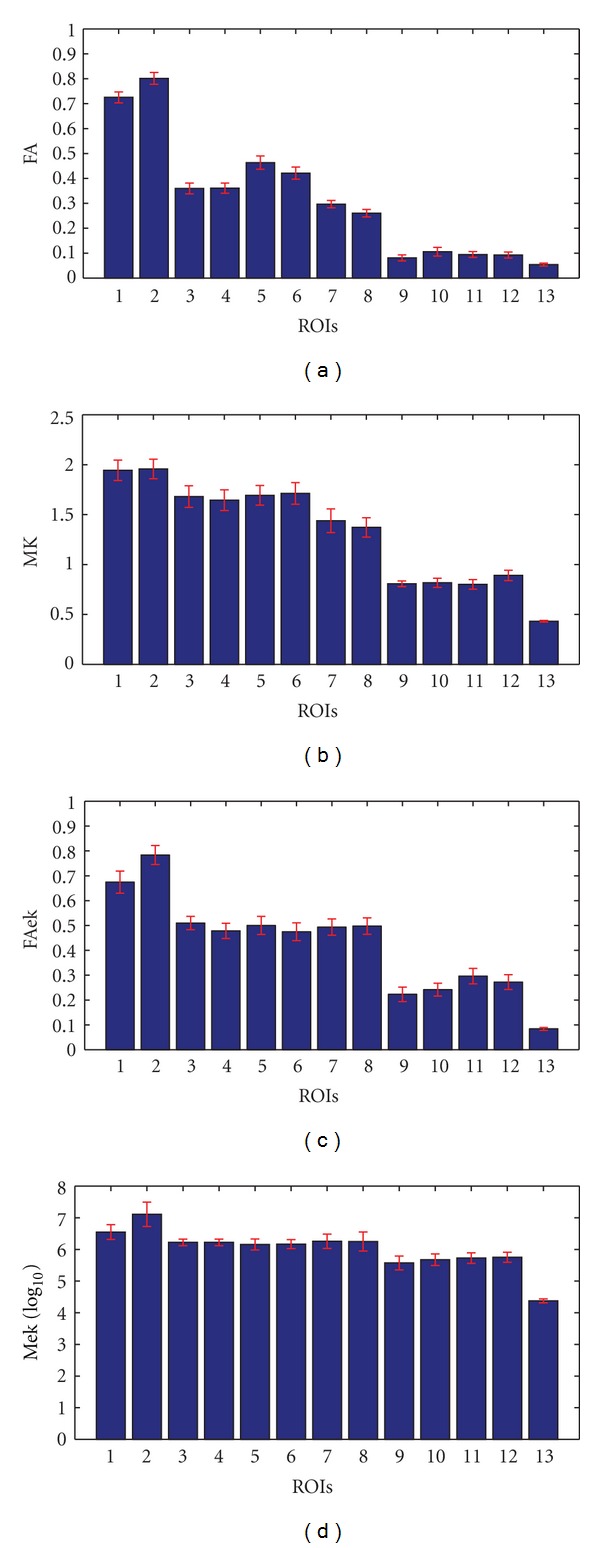
Mean variance of every ROIs, (1-2) the knee and splenial of callosum; (3–6) the crossing fibers; (7-8) the thalamus; (9–12) the cerebral cortexes; 13 is CSF.

The RIOs' statistics are figured in Figure 3. Different ROIs show pronounced average values (*P* < 0.05), and mainly five kinds of tissues in the ROIs. In a general view, the corpus callosum, cerebral cortex and CSF can be recognized obviously, while ((c)–(f)) cannot distinguish the crossing fiber tissues and thalamus. In detail, the first two ROIs, which are two parts of corpus callosum: the knee and the splenium, result in pronounced different values (*P* < 0.05). As the fibers in the splenium are mostly more slender than the knee, and its diffusion environment is more restricted or non-Gaussian. The MK shows similar values of the two parts (1.94 ± 0.15, 1.96 ± 0.14), but it is MK that can only differs from the crossing fiber (1.67 ± 0.16) and thalamus (1.42 ± 0.17) which is full of both cytons and fibers. The cerebral cortex, which mainly consists of cytons or cell membranes is which all parameters can obviously differ from white matter but the CSF in FA. What is special is that Mek is just showing the index number of the mean of  *D*-eigenvalues, as the values is very large and resulting in a lower gray contrast between different tissues.

Having a whole picture of these ROIs tissues' structure and considering the diffusion environment, we can select the freest and the most restricted: CSF and corpus callosum especially the splenium. Then the following crossing fiber area, basal ganglia (thalamus here) and cerebral cortex are less free successively. Freer the environment is, more Gaussian the Diffusion displacement distribution is. So, MK gives a good distinguish, but not very precise; FAek distinguishes different tissues more in details. Compared with AKCd, MK does not show stably to specific structure while the AKCd performances better.

**Figure 4:**
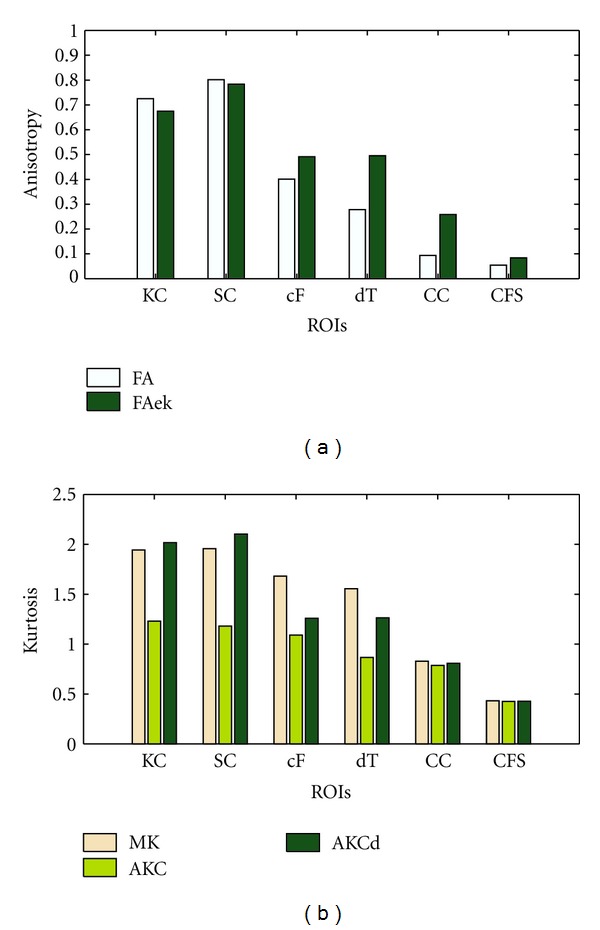
Anisotropy and kurtosis. In (a) several anisotropy values (FA, FAek) were detected averaging the volume value in different ROIs (knee of callosum, splenium callosum, crossing fiber, dorsal thalamus, cerebral cortex, CFS) as well as in (b) about kurtosis values; the statistical significance is all  *P* < 0.05.

Figure 4 gives a visualized comparison of different tissues about the same property (anisotropy and kurtosis) with specific method. From the figure, FAek also performances similarly with FA, but gains better contrast in cerebral cortex (0.28 ± 0.03). FAek shows high sensibility to gray matter as well as that like thalamus. Considering Figure 4(b), the kurtosis, MK shows low gray contrast, and AKCd and MK are better recognized, but MK shows a significant difference between crossing fibers and thalamus.

### 3.2. Results of Histogram Analysis

**Figure 5:**
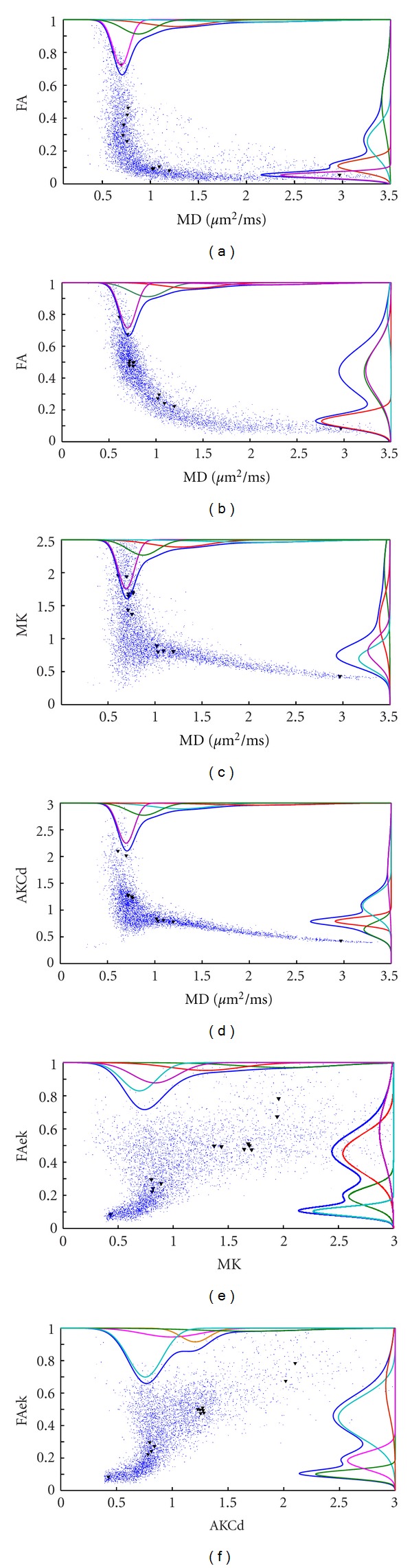
Scatterplot analysis. ((a)–(f)) give a visualized relationship between different parameter maps, and the Gaussian decomposition of the histogram is drew along the axis. Previous ROIs for different tissues' data were also marked on it.

With the principle that the gray values or parameters of the same characteristic tissues will be under a displacement of Gaussian function and independent from different tissues, the parametric map's histogram is decomposed using first-order Gaussian mixed signals. The mask was used in order to ignore the zeros background. MD map can gives a practical view of the tissues, so FA, FAek, Mek, and AKCd are compared with it in Figure 5, and also the kurtosis parameters' relativity are shown in Figures 5(e) and 5(f). 

In Figure 5(a), FA has a wide range when MD is low which represents white matter mainly, and MD shows also a wide range when FA is low which represent gray matter and CSF mainly. But there is no relativity between them and most information is distributed where both FA and MD are low. Following Figure 5(b), FAek has more balanced distribution of histogram, an obvious subpeak, so the most information distributes where higher FAek and low MD, which indicates more sensitive to white matter. And there has some negative correlation. In Figures 5(c) and 5(d), MK and AKCd have the similar distribution with MD, while MK shows more balanced with its MK value range is wider than AKCd. But in Figures 5(e) and 5(f), the decompositions of AKCd's histogram are much independent and signi orientation is transverseficant, while the MK's are much overlapped with others. As known that Mek has low gray contrast, and here it has an obvious negative correlation with MD in Figure 5(d), because the points are distributed along a similar line with MD increasing.

## 4. Discussion

As known theoretically, the DKI specifically the kurtosis tensor characterizes more detailed information and can show us a real insight into the microstructure of brain tissues. Practically, many work about kurtosis using DKI were carried out verifying that kurtosis provides more information and is more sensitive than diffusivity, but the kurtosis absolutely because of its complexity.

It is obvious that MK has great potential in biotissues mapping, such as tumor diagnosis and other Nerve damage disease, and kurtosis tensor can also provide an insight into the brain tissues' microstructure especially for white matter. These  *D*-eigenvalues of kurtosis indicate more information about tissues microstructure where diffusivity cannot. However, FAek performs much better than MEK, that is to say that these  *D*-eigenvalues can really indicate the complexity of the microenvironment but the average level of kurtosis is less sensitive. Here the cause maybe the number of the  *D*-eigenvalues or the algorithm. What is common and important is that all kurtosis information gives more information or sensitivity about white matter microstructures [3–8].

From the black triangles in Figures 5(a) and 5(b), we can see FA recognizing cortex and thalamus as the same, but FAek recognizes crossing fibers and thalamus as the same, and FAek differs from the cortex and CSF obviously. In Figures 5(e) and 5(f), the difference is that MK recognizes the thalamus and cortex which is better than AKCd.

 In the Figure 5(c), MK is the directly kurtosis calculation while the Mek is from  *D*-eigenvalues of kurtosis tensor, which the “*D*-” means diffusion in original paper [9]. That is to say MK is the real kurtosis information but not accurate, but Mek is the “diffusivity” information of kurtosis tensor, because there is a stronger relativity between Mek and MD. Here “diffusivity” means the level of 2-order diffusion coefficient. But it shows actually different evaluation of the tissues, maybe just the level of complexity. In the results other parameters' classification between the crossing fibers and thalamus show that they are likely the same complexity while not exactly the diffusivity in some degree, except MK. 

**Figure 6:**
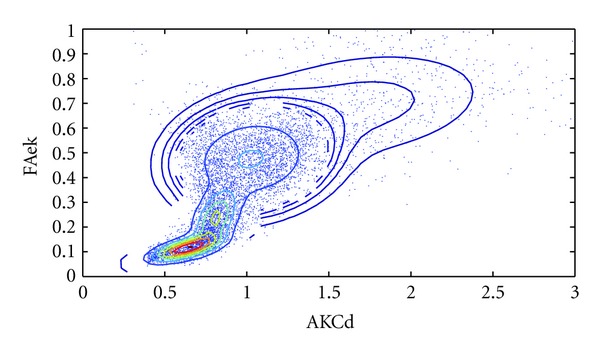
Scatter between AKCd and FAek, showing a 4 independent Gaussian distribution and probability gradients.

Kurtosis information can be used to get a more delicate classify of the tissues [12, 13]. The scatter figure and the Gaussian distribution classifying can give us ideas about image segmentation of different tissues. Within Figure 5 different two parameters put the tissues in different locations. In this part, FAek associated with AKCd and MD classified the typical tissues well. According to this, Figure 6 shows a different cluster of the scatter points.

In Figure 6, with previous knowledge, these 4 Gaussian distribution represent 4 kinds of tissues: CSF, Gray matter, crossing fibers; single diffusion orientation areas (corpus callosum). However, the third Gaussian class has a wide range which represents a collection of all crossing fiber areas as their complicated various fiber structures. 

For achieving better jobs, there are several aspects need be considered further. 

Firstly, the calculation of kurtosis anisotropy is just similar to DT. However, the  *D*-eigenvalues and vectors have no clear meaning, and even more important is that DT's three eigenvectors are always of orthogonal to each other while the  *D*-eigenvectors' orientation are random here. For example, same eigenvalues with different eigenvectors give different anisotropy properties. 

Secondly, these parameters in this paper are just aimed at the contrast or discrimination between different anatomical structures at the 2-D imaging level. Anyhow this work is a start, and what's need more is the white matter microstructure and 3D space reconstruction. Several studies about the partition of diffusion or ODF have carried out aiming at the 3D space diffusion property distribution [13, 14].

Thirdly, clearer relationship between DT and KT should be clearer and what to do with them should be clear. For example, how the 4-order statistic kurtosis is related practically to the 2-order variance which suggests thatdiffusion coefficient here should be clear. About this, popular speaking think the kurtosis can revise or make up the deviation from Gaussian, but not in a specific way. Another idea about this is that DKI gives the Gaussian diffusion and non-Gaussian diffusion. 

Finally, not least, the imaging processing to reducing noises or advancing SNR before fitting (2) is most important. Because high-order kurtosis is more sensitive to errors than diffusivity such as what appears as error spots in Figures 1(e) and 1(f) though using appropriate pre- and postprocessing. What is worse is that it will reduce the fitting results. So better signal processing is needed, or just increase the repeat times of signal acquisition (AVERAGE or NEX) but losing acquisition time (TA) for better signals from the view of experiment setting [15].

## 5. Conclusion

DKI is a straightforward extension of DW signals that provides a sensitive measurement of tissue structure by quantifying the non-Gaussian degree of water diffusion. DKI has been demonstrated to be highly sensitive and directionally specific in detecting brain maturation processes, and the parametric analysis of kurtosis tensor was carried out in this paper. The results indicated that more detailed insights of the microstructure can be detected and differed from diffusivity of diffusion tensor by the kurtosis tensor. 

The  *D*-eigenvalues of kurtosis tensor give the diffusivity information about tissues complexity which is different from diffusion coefficient, but the FAek (fractional anisotropy of these eigenvalues) shows more different properties than FA of diffusion tensor, which means the level of tissues complexity. Multiparameters analysis can give more detailed tissues of human brain. Diffusion kurtosis tensor can show a more comprehensive and sensitive detection of subtle difference, but more energy should be paied for this kurtosis tensor.
